# Asthma as a Modifiable Risk Factor for Dementia and Neurodegeneration: A Systematic Review

**DOI:** 10.7759/cureus.77540

**Published:** 2025-01-16

**Authors:** Mateusz Szczerba, Aleksandra Hejnosz, Gabriel Majewski, Olga Grodzka, Izabela Domitrz

**Affiliations:** 1 Department of Neurology, Medical University of Warsaw, Warsaw, POL

**Keywords:** alzheimer’s disease, asthma, chronic inflammation, dementia, neurodegeneration

## Abstract

Asthma remains a significant health concern, with increasing incidence rates and a notable annual mortality rate. Although the pathophysiology of asthma and its impact on the respiratory system are well understood, leading to effective symptom control, the broader effects of chronic localized airway inflammation on the rest of the body remain less clear. One potential consequence of this persistent state is an elevated risk of developing dementia across various etiologies, including Alzheimer’s disease (AD). This systematic review aims to elucidate the underlying mechanisms driving this association, with a particular focus on identifying neurodegeneration markers detectable through laboratory and imaging studies. Additionally, it examines the impact of asthma treatments on this potential risk, exploring their possible contributory role in the pathogenesis of dementia. Such insights should inform the development of personalized treatment strategies for asthma patients, aimed at preventing or delaying the onset of dementia. However, further research is necessary to understand the underlying mechanisms of the connection fully and crucial to integrate it with routine clinical practice.

## Introduction and background

Asthma is defined as a respiratory condition with chronic inflammation of the lungs that leads to structural changes in the airways and varying degrees of airflow obstruction. Key symptoms include dyspnea, cough, chest constriction, and wheezing. Depending on disease severity and course, it can manifest episodically or persistently, with clinical presentations ranging from acute exacerbations to chronic, stable phases. [1]. According to the latest global estimates, asthma prevalence reached 262 million cases in 2019 and caused 455,000 deaths [2]. Although it is commonly associated with childhood diseases, asthma poses a concern in every age group, even among the elderly [3]. Observations and epidemiological data have increasingly identified asthma as a potential contributor to the development of other conditions, including cardiovascular diseases, metabolic syndrome, and even mental health disorders [4-6]. Recently, there has been growing interest in exploring the link between asthma and dementia across various etiologies. Research with animal studies has shown that local airway inflammation can trigger systemic inflammation with pathological microglial reaction that eventually leads to neurodegeneration [7]. 

A major neurocognitive disorder is characterized by a significant decline in at least one domain of cognition, such as executive function, complex attention, language, learning, memory, perceptual-motor skills, or social cognition [8]. Dementia can result from various etiologies, including vascular disease, frontotemporal lobar degeneration, Lewy body disease, Parkinson's disease, infections, Huntington's disease, prion diseases, trauma, or medication use. Often, multiple causes contribute to the manifestation of dementia in patients [9]. However, the most common cause remains Alzheimer's disease (AD) [10]. This focus on the asthma-dementia link is particularly crucial as dementia continues to pose a significant challenge in public healthcare worldwide, underscoring the need for effective strategies to prevent its progression [11]. Understanding the potential connection between asthma and dementia is not only important for early intervention but also for developing comprehensive treatment plans that address both respiratory and cognitive health, especially in terms of hypoxia as a factor that can impact higher cortical functions and contribute to cognitive symptoms.

This review aims to summarize the scientific literature from the past five years, providing an overview of the current understanding of the relationship between asthma and dementia, including epidemiology, key observations, potential causes, and future directions.

## Review

Search strategy

An examination of current literature on the relationship between asthma and dementia has identified the main areas that will be explored in this review. The focus of the paper is on the association between asthma and dementia, particularly concerning their epidemiology, underlying mechanisms, biomarkers of neurodegeneration, and treatment approaches. These topics have shaped the framework of the review, enabling a thorough analysis of the relevant research and the integration of findings related to the issues at hand. To capture all potential associations, we included the term "Alzheimer's disease" in our search, as it is the most common cause of dementia. Finally, the search process involved combining the terms "asthma," "dementia," and "Alzheimer’s disease," using the search string: ("asthma") AND ("dementia" OR "Alzheimer’s disease"). All these terms are indexed within Medical Subject Headings (MeSH), the controlled vocabulary thesaurus maintained by the National Library of Medicine for categorizing articles in PubMed.

This search was conducted in July 2024 using the PubMed and Excerpta Medica database (Embase) databases. To focus on the most recent findings, we limited the scope of the review to publications from January 2019 to July 2024. This method produced 1,646 records (283 from PubMed and 1,363 from Embase).

Study selection

After the initial search, a total of 1,646 collected articles were examined for potential duplicates, leading to the detection and exclusion of 207 duplicates. A subsequent screening process was conducted to ensure alignment with the review’s themes and structure. This review adhered to the Preferred Reporting Items for Systematic Reviews and Meta-Analysis (PRISMA) 2020 guidelines [12], ensuring a consistent and rigorous methodology.

Based on the main focus of the review, screening was guided by criteria established by the co-authors. The assessment focused on whether each article addressed asthma, dementia, or AD, as well as the relevance of the content to the review’s established themes. The inclusion criteria comprised all observational studies (including prospective or retrospective cohort, case-control, cross-sectional, case series, and case study designs) and experimental studies (randomized controlled trials). Only articles published in English were considered.

**Figure 1:**
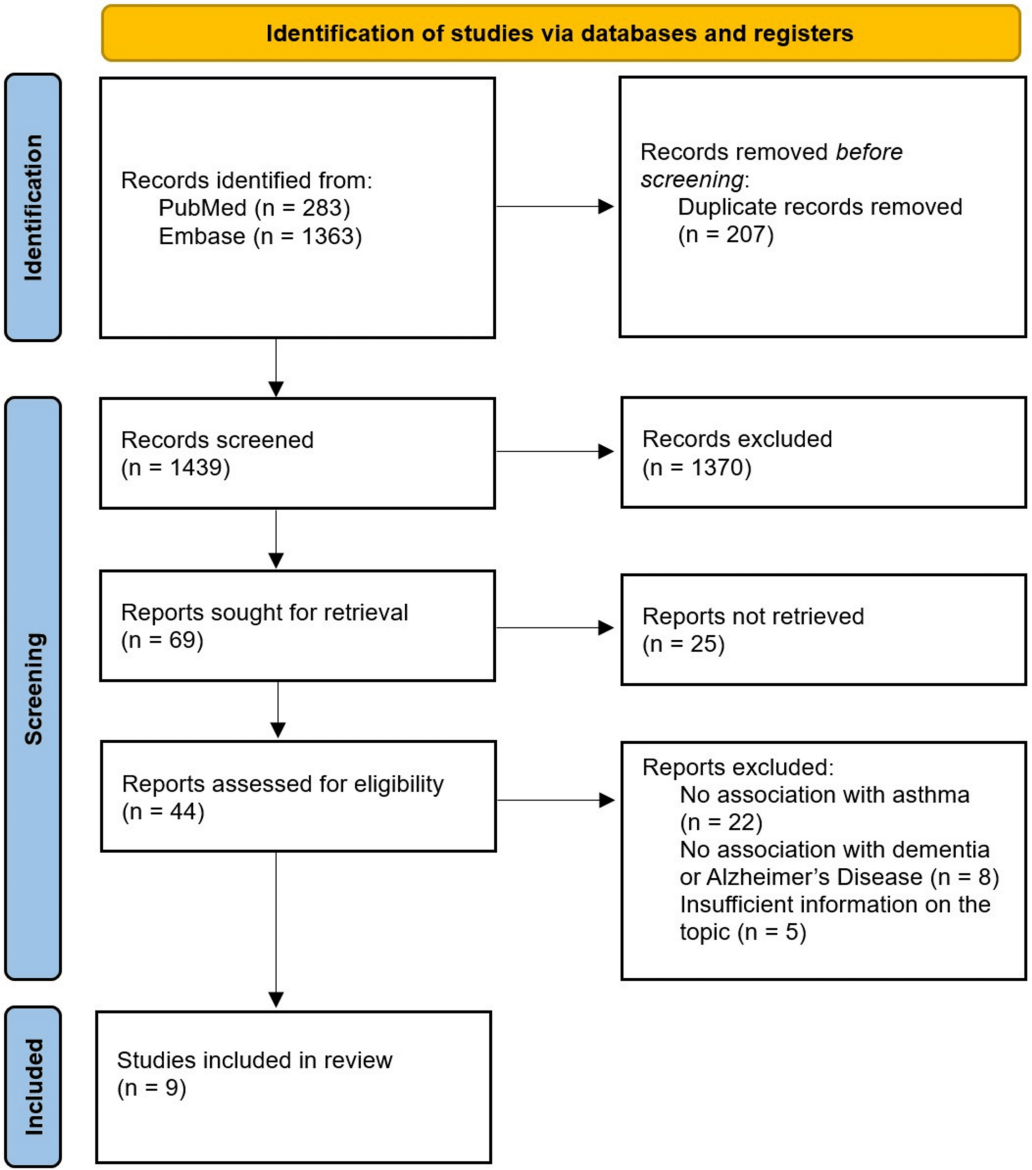
Preferred Reporting Items for Systematic Reviews and Meta-Analyses (PRISMA) flowchart for the article selection process n: number of studies; Embase: Excerpta Medica database Reference: [12]

In the first stage, details of the 1439 records were organized in Microsoft Excel (Microsoft Corp., Redmond, WA), with data on titles, sources, and study types. Then, co-authors reviewed titles for consistency with the criteria, resulting in an exclusion of 1,370 articles and 69 articles qualifying for the next stage of screening. This process was repeated for abstracts, leading to the rejection of 25 articles at this stage. A total of 44 articles deemed relevant, along with any conflicting assessments, were subjected to full-text screening and subsequently examined to determine their eligibility. Additionally, 35 research papers were excluded due to reasons such as lack of association with asthma or dementia and insufficient information on the topic. Any differences of opinion among the reviewers regarding inclusion or exclusion decisions at any stage of the study selection process were resolved by re-evaluating the article and discussing it collectively among all authors to reach a consensus. If no agreement could be reached, the final decision was made by the study supervisor. This process ultimately narrowed the selection to nine studies included in this review. A flow diagram shows the entire process of selection (Figure 1). Two authors extracted data from each of the reports included in the review, organizing them into a spreadsheet. The process was then reviewed and summarized by a third author.

Critical appraisal tool and risk of bias evaluation

The evaluation of the risk of bias was carried out independently by two reviewers and compared. Given the variety of study designs included in the review, it was necessary to employ two distinct assessment tools. Specifically, eight studies were evaluated using the National Institutes of Health (NIH) Quality Assessment Tool for Observational Cohort and Cross-sectional Studies [13], while one study was appraised with the NIH Quality Assessment Tool for Case-Control Studies [13]. Any discrepancies in the bias assessments were resolved through thorough discussions among all reviewers to achieve consensus. The articles were classified as either good or fair, reflecting a low risk of bias (Appendices A,B). This classification enabled the inclusion of data from all evaluated studies in the subsequent discussion.

Exploring the controversial link between asthma and dementia

The systematic increase in life expectancy is contributing to a growing prevalence of dementia and AD, posing significant challenges to healthcare systems [14]. Consequently, there is an increasing need to identify risk factors associated with these conditions. In recent years, attention has been focused on the co-occurrence of allergies, including asthma, and various forms of dementia, to develop optimal public health prevention strategies and effective treatments [15].

Bartels et al., in their analysis of a cohort comprising 5,460,732 Medicare beneficiaries in the United States of America, demonstrated that individuals with asthma had a 20% to 33% higher risk of developing AD within two years compared to those without asthma [16]. The prevalence of AD was 25% to 48% higher in patients with asthma. Furthermore, patients with asthma exhibited a higher prevalence of general dementia (20.3%) compared to those without asthma (12.9%). However, the authors of the study noted an uneven distribution of socioeconomic factors within the study population, which may have influenced the results [16]. These findings suggest that asthma could be a significant risk factor for the development of dementia and AD.

Supporting this, a 2023 study examined the impact of allergic diseases (including asthma, allergic rhinitis, and atopic dermatitis) on the risk of dementia. In this study, asthma was positively correlated with an increased risk of dementia (hazard ratio (HR) 1.20; 95% CI 1.19-1.22), including AD (HR 1.22; 95% CI 1.20-1.24) and vascular dementia (HR 1.09; 95% CI 1.05-1.14). Additionally, the risk increased with the number of comorbid allergic diseases, with the strongest association observed in individuals aged 50-64 years [17]. The authors of both studies suggest that the common pathomechanism linking these conditions may be chronic inflammation, which can lead to systemic changes through local effects [16,17].

Conversely, different results were reported by Kim et al. in a 2019 study [18]. The prevalence of asthma in dementia cases was 22.6%, which was not significantly different from the control group, where it was 22.3%. These findings did not show a statistically significant association between asthma and dementia, regardless of subgroups analyzed by gender and patient age (<80 years old and >80 years old). In this study, the impact of other respiratory diseases, including chronic obstructive pulmonary disease (COPD), was minimized by including them as variables in the statistical analysis. Although asthma is associated with an increased risk of inflammatory conditions such as ischemic heart disease and diabetes, their impact was also accounted for, which may have contributed to the lack of a significant association between asthma and dementia [18]. These findings contrast with previously discussed studies on the association between asthma and dementia risk [16,17].

Thus, there is variability in the results of studies published over the last five years. Further research is needed to better understand and explain these discrepancies and to precisely determine the extent to which asthma may influence the risk of developing dementia, including AD.

Biomarkers of neurodegeneration and synaptic damage

Several studies have identified asthma as a potential risk factor for dementia, particularly AD [15,19]. There has been a growing interest in exploring physical brain abnormalities that might reflect asthma's influence on brain structure and consolidation. A study by Nair et al. focused on the connection between asthma and dementia by analyzing cognitive performance and cerebrospinal fluid (CSF) biomarkers in 60 patients with and 315 patients without asthma [20]. The results proved severe asthma to be associated with elevated levels of neurogranin, a biomarker indicative of synaptic degeneration, and alpha-synuclein linked to neurodegeneration. Quantitative analyses revealed that neurogranin concentrations in patients with severe asthma were elevated by 147% compared to patients with mild asthma. Additionally, neurogranin levels were found to be 123% higher in severe asthma patients than in non-asthmatic controls. Elevated neurogranin levels, which have high specificity for AD, indicate that severe asthma might specifically increase the risk of AD-related dementia [20,21]. Nevertheless, severe asthma was also associated with higher concentrations of alpha-synuclein, suggesting a broader impact on neurodegeneration. Comparing biomarker results to the controls, we may conclude that asthma contributes to the risk of dementia by accelerating neuron and synaptic damage. Other results suggest that severe asthma may amplify dementia risk, especially by compounding cardiovascular risks, genetic predispositions, and biomarkers linked to cognitive decline, like tau and amyloid proteins. Although it primarily associates with mechanisms relevant to AD, it also points to a broader impact on general neurodegeneration​ [21]. However, the relationship between vascular dementia and AD in the context of severe asthma requires further investigation to fully understand these links. Another single-center study analyzed the data from 31 asthma and 186 non-asthma patients with a family history of AD. Similarly to the previous study, diffusion magnetic resonance imaging (dMRI) and CSF analysis were conducted. Asthma patients showed stronger associations between white matter abnormalities and markers of AD pathology, such as Aβ42/Aβ40 and phosphorylated tau (phospho-tau181P), compared to controls. Asthma patients presented lower Aβ42/Aβ40 levels that were associated with lower Neurite Density Index (NDI) and fractional anisotropy (FA), which are indicators of the integrity of white matter structures in the brain, with higher values suggesting well-organized and healthy neural fiber tracts. They also had higher mean diffusivity (MD) and radial diffusivity (RD), which might indicate neurodegeneration [22]. These associations remained significant even after controlling for cardiovascular disease and apolipoprotein E ε4 allele (APOE4) genetic risk factors, indicating that the effects of asthma on brain microstructure are at least partially independent of these factors [20]. A study by Rosenkranz et al. revealed that higher concentrations of glial fibrillary acidic protein, a marker of neuroinflammation, were associated with greater white matter deterioration in asthma patients. Neurofilament light chain, a marker of neurodegeneration, also showed a relationship with white matter changes [23].

Structural changes of the brain and cognitive decline

Previously mentioned research by Rosenkrantz et al. (2022) investigated the impact of asthma on brain structure, neurodegeneration, and cognitive function in 111 participants with asthma and 135 non-asthmatic controls. The study found significant structural differences in white matter between asthma patients and healthy controls, with more progressed deterioration in asthma-positive patients [23].

The study used dMRI to assess white matter integrity in asthma patients. The findings revealed widespread and significant differences in white matter microstructure between individuals with asthma and healthy controls, with more severe deterioration in those with severe asthma. Specifically, asthma patients presented greater deterioration in white matter integrity, with more pronounced changes in regions such as the corticospinal tract, external capsule, inferior and superior longitudinal fasciculi, and inferior frontal-occipital fasciculi. These changes were particularly associated with regions of the brain involved in cognitive functions, such as the longitudinal fasciculi, and were correlated with a marginal decline in processing speed.

The relationship between asthma severity and white matter deterioration highlights the potential impact of asthma on cognitive function. Despite ongoing asthma treatment, structural brain changes persisted, suggesting that inflammation not controlled by corticosteroids may contribute to neurodegeneration and, consequently, cognitive decline. The study of Nair et al. observed that asthma patients exhibited more pronounced white matter deterioration, particularly in regions like the corticospinal tract, superior longitudinal fasciculus, and external capsule regions [20]. The study also found that asthma amplified the typical age-related decline in white matter integrity with increased MD and RD in regions like the corpus callosum and internal capsule. It is suggested that asthma may exacerbate the natural aging process in the brain, leading to earlier and more severe cognitive deficits [21].

**Figure 2:**
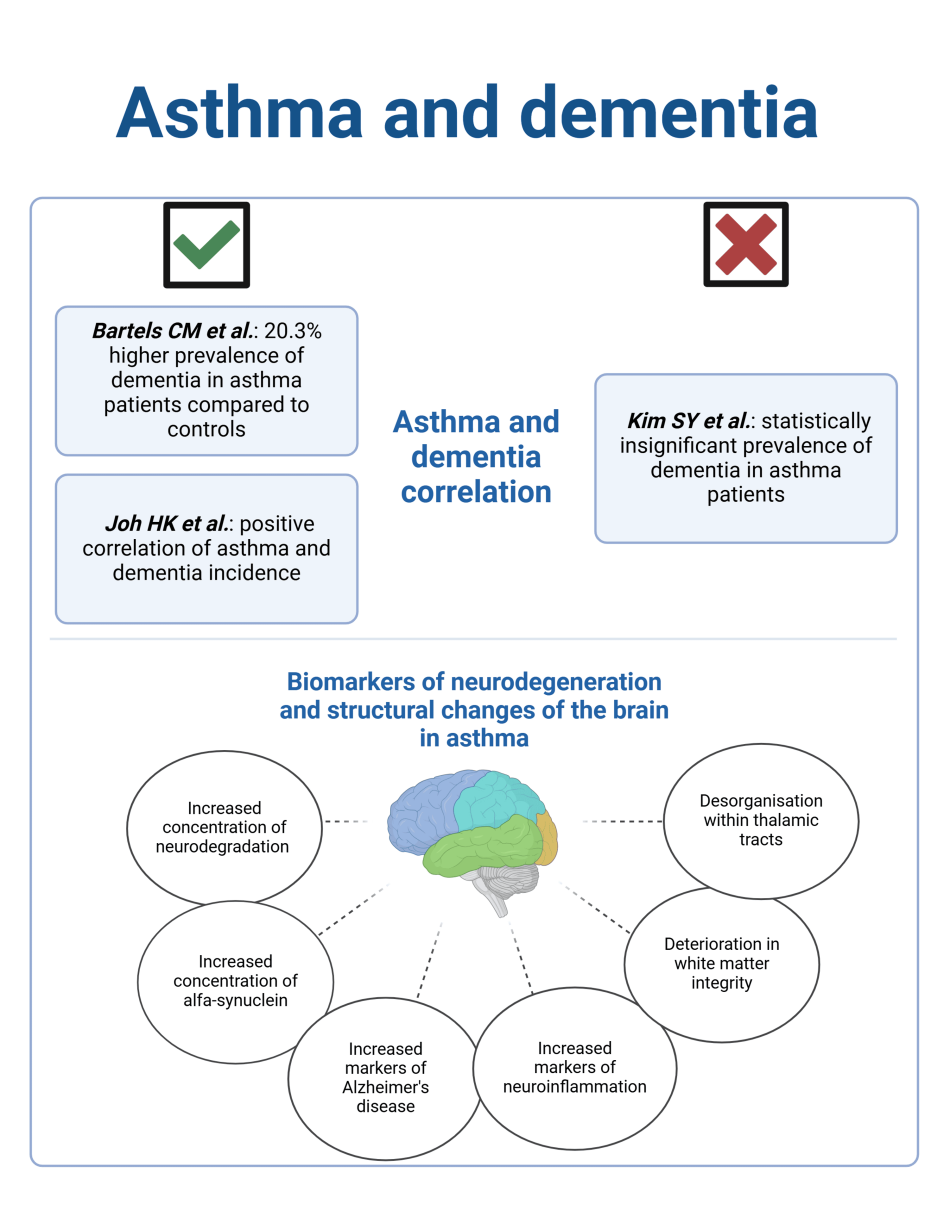
The latest reports on the connection between asthma and the occurrence of dementia and the potential impact of asthma on the brain are reflected in neuronal biomarkers and imaging. References: [16-18,20,21,23,24] Created by the authors in BioRender. (Created by the authors in BioRender. Hejnosz, A.(2025) https://BioRender.com/n86h859. Reproduced with permission from the original publisher.

It was further highlighted that asthma was associated with stronger relationships between dMRI metrics and preclinical Alzheimer’s cognitive composite (PACC) slopes, which is a measure of cognitive decline. Lower NDI and FA were associated with more negative PACC slopes in asthma patients, indicating accelerated cognitive deterioration over time. The researchers suggested structural changes observed in the brains of asthma patients may have significant implications for cognitive function, particularly in terms of processing speed and executive function [20]. Some of these conclusions were mentioned in the more recent study of Wang et al. [24]. 31 patients with bronchial asthma and 31 controls were recruited for MRI scans and resting-state functional MRI (rs-fMRI). The main focus was dedicated to the thalamic functional connectivity (FC) between the two groups. It was found that patients with bronchial asthma presented abnormal changes in altered FC in functional MRI (fMRI) compared to controls. Patients with asthma exhibited increased FC between the left thalamus and regions such as the left cerebellar posterior lobe, left postcentral gyrus, and right superior frontal gyrus. Decreased FC was observed between the thalamus and areas like the lentiform nucleus and the left corpus callosum. It is postulated that it may result from potential compensatory mechanisms or inadequate brain reorganization. These patterns also seemed to influence the cognitive impairment and psychiatric aspects of the patients. The FC between the left thalamus and right superior frontal gyrus showed a negative correlation with cognitive scores and a positive correlation with depression scores. The results highlight that asthma in patients might influence a wide range of functions, including sensory processing, cognitive control, emotional regulation, and motor coordination [24]. Overall biomarkers of neurodegeneration and structural changes of the brain are presented in Figure 2.

The role of pharmacotherapy in asthma and dementia

Currently, the most effective approach in the fight against dementia is prevention. To develop optimal prevention strategies, it is essential to precisely study the impact of asthma pharmacotherapy on risk factors for the development of dementia, including AD. Given the complexity of the pathomechanisms of these conditions, it is also important to understand the role of pharmacotherapy when asthma co-occurs with dementia or, specifically, AD. A study conducted by Joh et al. analyzed the relationship between asthma therapy, which, as previously mentioned, influences the pathophysiology of both conditions and the risk of developing dementia [17]. The results indicated that the use of systemic (± topical) corticosteroids was associated with a significantly higher risk of overall dementia (HR=1.25, 95% CI=1.23-1.27) and AD (HR=1.27, 95% CI=1.25-1.29) compared to those without asthma. Additionally, the use of first-generation antihistamines increased the risk of developing dementia in patients with asthma compared to those without asthma. Importantly, patients with asthma who were not treated with corticosteroids or first-generation antihistamines also showed an increased risk of developing dementia. This suggests that, regardless of the treatment used, asthma may lead to an increased risk of developing dementia. It was not until the use of second-generation antihistamines that the risk of developing dementia and AD was reduced to a level comparable to those without a diagnosis of asthma [17].

Similar findings were reported by Ishikura et al., who studied the effects of leukotriene receptor antagonists (LTRAs) in patients with asthma [25]. The use of LTRAs led to a 58% reduction in the risk of developing dementia compared to a control group that did not use LTRAs (adjusted HR=0.42, 95% CI=0.20-0.87, p=0.019). These results suggest that LTRAs may have potential use as preventive drugs in the context of dementia. Lampela et al. analyzed the treatment structure of patients with asthma/COPD and AD [26]. The co-occurrence of these diseases resulted in a less frequent use of acetylcholinesterase inhibitors as first-line treatment compared to patients without asthma/COPD. Instead, memantine was used more frequently. The study authors suggested that, in addition to differences in the stage of AD, this may be due to an attempt to prevent complications arising from the simultaneous use of two classes of drugs with opposite mechanisms of action. Given the increased risk of mortality in patients with asthma/COPD compared to those without these conditions, the choice of treatment in this group should be based on an individual assessment of the patient’s condition and the stage of AD [26]. These studies underscore the importance of the appropriate selection of asthma therapy in the context of dementia and AD prevention while highlighting the potential benefits and risks associated with different classes of drugs.

Limitations

The study faced several limitations. The research was conducted using a relatively small number of databases for identifying potentially relevant studies. Additionally, the literature review was restricted to studies published within the last five years and only in English. The data search was not conducted independently by each of the two co-authors. The analysis did not differentiate between various asthma subtypes and etiologies of dementia. Moreover, the studies did not consider other lifestyle factors, such as smoking, diet, physical activity, or environmental exposures.

## Conclusions

In recent years, there has been increasing attention on the potential mechanisms leading to the development of dementia. It appears that local inflammation associated with asthma may extend to the central nervous system, contributing to neurodegeneration, synaptic damage, and neuroinflammation, as reflected in imaging observations. Understanding the specific pathways through which asthma contributes to neurodegeneration, as well as the role of genetic predispositions and co-occurring cardiovascular risks, is crucial for developing targeted interventions that could prevent or delay the onset of dementia in asthma patients. This underscores the need for a comprehensive approach to patient care, potentially by designing asthma and dementia treatment for individual patients, especially in terms of different drug classes.

Identifying the most effective timing for therapeutic interventions is especially crucial given the rising prevalence of dementia. Future research should explore the mechanistic pathways connecting asthma and dementia and investigate therapeutic options that may slow or prevent cognitive decline. It is also essential to consider the effects of hypoxic episodes during asthma attacks, as they may result in ischemic changes in the brain, leading to secondary neurodegeneration.

## Appendices

Appendix A 

**Table 1:** Quality-assessment tool for observational cohort and cross-sectional studies (National Institutes of Health) (+) yes/low risk; (-) no/high risk; NA: not applicable; Q: question Q1. Was the research question or objective in this paper clearly stated? Q2. Was the study population clearly specified and defined? Q3. Was the participation rate of eligible persons at least 50%? Q4. Were all the subjects selected or recruited from the same or similar populations (including the same time period)? Were inclusion and exclusion criteria for being in the study prespecified and applied uniformly to all participants? Q5. Was a sample size justification, power description, or variance and effect estimates provided? Q6. For the analyses in this paper, were the exposure(s) of interest measured prior to the outcome(s) being measured? Q7. Was the timeframe sufficient so that one could reasonably expect to see an association between exposure and outcome if it existed? Q8. For exposures that can vary in amount or level, did the study examine different levels of the exposure as related to the outcome (e.g., categories of exposure, or exposure measured as continuous variable)? Q9. Were the exposure measures (independent variables) clearly defined, valid, reliable, and implemented consistently across all study participants? Q10. Was the exposure(s) assessed more than once over time? Q11. Were the outcome measures (dependent variables) clearly defined, valid, reliable, and implemented consistently across all study participants? Q12. Were the outcome assessors blinded to the exposure status of participants? Q13. Was loss to follow-up after baseline 20% or less? Q14. Were key potential confounding variables measured and adjusted statistically for their impact on the relationship between exposure(s) and outcome(s)? Reference: [13]

References	Q1	Q2	Q3	Q4	Q5	Q6	Q7	Q8	Q9	Q10	Q11	Q12	Q13	Q14	Overall rating
Bartels et al. [16]	+	+	+	+	+	+	+	+	+	-	+	-	+	+	Good
Joh et al. [17]	+	+	+	+	+	+	+	+	+	-	+	-	+	+	Good
Nair et al. [20]	+	+	+	+	-	+	+	-	+	-	+	-	+	+	Fair
Nair et al. [21]	+	+	+	+	-	+	+	+	+	-	+	-	+	+	Good
Rosenkranz et al. [23]	+	+	+	+	-	+	NA	+	+	-	+	+	NA	+	Good
Wang et al. [24]	+	+	+	+	-	+	NA	-	+	-	+	-	NA	+	Fair
Ishikura et al. [25]	+	+	+	+	-	+	+	-	+	-	+	-	+	+	Fair
Lampela et al. [26]	+	+	+	+	-	+	+	+	+	-	+	-	+	+	Good

Appendix B 

**Table 2:** Quality-assessment tool for case-control studies (National Institutes of Health) (+) yes/low risk, (-) no/high risk; Q: question Q1. Was the research question or objective in this paper clearly stated and appropriate? Q2. Was the study population clearly specified and defined? Q3. Did the authors include a sample size justification? Q4. Were controls selected or recruited from the same or similar population that gave rise to the cases (including the same timeframe)? Q5. Were the definitions, inclusion and exclusion criteria, algorithms or processes used to identify or select cases and controls valid, reliable, and implemented consistently across all study participants? Q6. Were the cases clearly defined and differentiated from controls? Q7. If less than 100 percent of eligible cases and/or controls were selected for the study, were the cases and/or controls randomly selected from those eligible? Q8. Was there use of concurrent controls? Q9. Were the investigators able to confirm that the exposure/risk occurred prior to the development of the condition or event that defined a participant as a case? Q10. Were the measures of exposure/risk clearly defined, valid, reliable, and implemented consistently (including the same time period) across all study participants? Q11. Were the assessors of exposure/risk blinded to the case or control status of participants? Q12. Were key potential confounding variables measured and adjusted statistically in the analyses? If matching was used, did the investigators account for matching during the study analysis? Reference: [13]

References	Q1	Q2	Q3	Q4	Q5	Q6	Q7	Q8	Q9	Q10	Q11	Q12	Overall rating
Kim et al. [18]	+	+	+	+	+	+	-	+	-	-	+	+	Good

## References

[1] Maslan J, Mims JW (2014). What is asthma? Pathophysiology, demographics, and health care costs. Otolaryngol Clin North Am.

[2] GBD 2019 Diseases and Injuries Collaborators (2020). Global burden of 369 diseases and injuries in 204 countries and territories, 1990-2019: a systematic analysis for the Global Burden of Disease Study 2019. Lancet.

[3] Hisinger-Mölkänen H, Honkamäki J, Kankaanranta H (2022). Age at asthma diagnosis is related to prevalence and characteristics of asthma symptoms. World Allergy Organ J.

[4] Pollevick ME, Xu KY, Mhango G (2021). The relationship between asthma and cardiovascular disease: an examination of the Framingham Offspring Study. Chest.

[5] Park S, Choi NK, Kim S, Lee CH (2018). The relationship between metabolic syndrome and asthma in the elderly. Sci Rep.

[6] Caulfield JI (2021). Anxiety, depression, and asthma: new perspectives and approaches for psychoneuroimmunology research. Brain Behav Immun Health.

[7] Hoogland IC, Houbolt C, van Westerloo DJ, van Gool WA, van de Beek D (2015). Systemic inflammation and microglial activation: systematic review of animal experiments. J Neuroinflammation.

[8] McKhann GM, Knopman DS, Chertkow H (2011). The diagnosis of dementia due to Alzheimer's disease: recommendations from the National Institute on Aging-Alzheimer's Association workgroups on diagnostic guidelines for Alzheimer's disease. Alzheimers Dement.

[9] Hugo J, Ganguli M (2014). Dementia and cognitive impairment: epidemiology, diagnosis, and treatment. Clin Geriatr Med.

[10] Reitz C, Mayeux R (2014). Alzheimer disease: epidemiology, diagnostic criteria, risk factors and biomarkers. Biochem Pharmacol.

[11] Stephan BC, Birdi R, Tang EY (2018). Secular trends in dementia prevalence and incidence worldwide: a systematic review. J Alzheimers Dis.

[12] Page MJ, McKenzie JE, Bossuyt PM (2021). The PRISMA 2020 statement: an updated guideline for reporting systematic reviews. Syst Rev.

[13] (2024). Study quality assessment tools. https://www.nhlbi.nih.gov/health-topics/study-quality-assessment-tools.

[14] Brookmeyer R, Johnson E, Ziegler-Graham K, Arrighi HM (2007). Forecasting the global burden of Alzheimer's disease. Alzheimers Dement.

[15] Chen MH, Li CT, Tsai CF (2014). Risk of dementia among patients with asthma: a nationwide longitudinal study. J Am Med Dir Assoc.

[16] Bartels CM, Chen Y, Powell WR (2024). Alzheimer incidence and prevalence with and without asthma: a Medicare cohort study. J Allergy Clin Immunol.

[17] Joh HK, Kwon H, Son KY (2023). Allergic diseases and risk of incident dementia and Alzheimer’s disease. Ann Neurol.

[18] Kim SY, Min C, Oh DJ, Choi HG (2019). Risk of neurodegenerative dementia in asthma patients: a nested case-control study using a national sample cohort. BMJ Open.

[19] Peng YH, Wu BR, Su CH, Liao WC, Muo CH, Hsia TC, Kao CH (2015). Adult asthma increases dementia risk: a nationwide cohort study. J Epidemiol Community Health.

[20] Nair AK, Van Hulle CA, Bendlin BB (2023). Impact of asthma on the brain: evidence from diffusion MRI, CSF biomarkers and cognitive decline. Brain Commun.

[21] Nair AK, Van Hulle CA, Bendlin BB (2022). Asthma amplifies dementia risk: Evidence from CSF biomarkers and cognitive decline. Alzheimers Dement (N Y).

[22] Salat DH (2014). Chapter 12: diffusion tensor imaging in the study of aging and age-associated neural disease. Diffusion MRI (Second Edition).

[23] Rosenkranz MA, Dean DC 3rd, Bendlin BB (2022). Neuroimaging and biomarker evidence of neurodegeneration in asthma. J Allergy Clin Immunol.

[24] Wang T, Huang X, Dai LX, Zhan KM, Wang J (2024). Functional connectivity alterations in the thalamus among patients with bronchial asthma. Front Neurol.

[25] Ishikura Y, Maeda-Minami A, Hosokawa M (2021). Leukotriene receptor antagonist use and dementia risk in patients with asthma: a retrospective cohort study. In Vivo.

[26] Lampela P, Tolppanen AM, Koponen M, Tanskanen A, Tiihonen J, Hartikainen S, Taipale H (2020). Asthma and chronic obstructive pulmonary disease as a comorbidity and association with the choice of antidementia medication among persons with Alzheimer’s disease. J Alzheimers Dis.

